# Suppurative Cervicomediastinitis From the Perspective of the Head and Neck Surgeon in a Tertiary Treatment Unit

**DOI:** 10.7759/cureus.67912

**Published:** 2024-08-27

**Authors:** Daniela Vrinceanu, Mihai Dumitru, Bogdan Banica, Oana Maria Patrascu, Mihaela Pertea, Mihai Radulescu, Andreea Marinescu

**Affiliations:** 1 ENT Department, Carol Davila University of Medicine and Pharmacy, Bucharest, ROU; 2 Oral and Maxillofacial Surgery Department, Bucharest University Emergency Hospital, Bucharest, ROU; 3 Pathology Department, Carol Davila University of Medicine and Pharmacy, Bucharest, ROU; 4 Department of Surgery 1, Grigore T Popa University of Medicine and Pharmacy, Iasi, ROU; 5 Thoracic Surgery Department, Carol Davila University of Medicine and Pharmacy, Bucharest, ROU; 6 Radiology and Imaging Department, University Emergency Hospital of Bucharest, Bucharest, ROU

**Keywords:** multidisciplinary approach, surgery, mediastinitis, infection, deep neck spaces

## Abstract

Introduction

Cervical suppurations represent an emergency pathology, with a dramatic evolution in the absence of adequate treatment. It frequently affects young people, hence the medico-legal implications of these cases. The anatomical substrate for the development of these deep cervical suppurations is represented by the cervical fascia and spaces. A distinct and extremely serious sub-chapter within diffuse cervical suppurations is necrotic cervical fasciitis, a polymicrobial infection with the most common oropharyngeal or odontogenic starting point, with rapidly progressive, destructive evolution in the deep fascial planes of the neck.

Materials and method

We will present a retrospective clinical study carried out on 26 cases diagnosed and treated between September 2013 and September 2018 in the ENT Clinic Department of the Bucharest University Emergency Hospital.

Results

Our retrospective analysis of a cohort of 26 patients in a tertiary referral center showed that deep cervical suppurations are slightly more common in men than in women. The most affected age groups were 50-59 years, followed by 20-29 years, representing a percentage of 53.84% of all cases. Also, 53.84% of the studied patients with deep cervical suppurations had a precarious and modest status. The most common clinical signs at presentation were malaise, cervical swelling, neck pain, dysphagia, fever, dysphonia, dyspnea, and cervical erythema. More than 60% of suppurations were odontogenic and 23% were caused by a traumatic element. Diabetes mellitus represents a comorbidity in 30.8% of patients, while 42.3% of patients had no personal pathological history, and thus this pathology has a lethal potential also in a patient in full health. In the study group, 46 (15%) had cervicomediastinitis, and 61.53% developed necrotizing fasciitis. One-third (34.61%) of our patients had undergone previous drainage surgery. Bacteriological examinations of the wound were with group C, D, G betahemolytic streptococcus, while 61.53% of the cultures were negative. Most patients required at least two cervicotomies. The average duration of hospitalization was 28.26 days, and the mortality rate was 23.07%; therefore, practically, one out of four cases resulted in death. In the studied group, no direct relationship can be established between the length of hospitalization and the favorable and unfavorable evolution of the patient. We propose a 10-step management protocol for the management of a cervical suppuration.

Conclusion

The multidisciplinary approach to these suppurations by the head and neck surgeon, the thoracic surgeon, the oromaxillofacial surgeon, anesthetist, imagist, specialist in infectious diseases, pathologist, psychologist, and so on, is the key to success in a patient who presents not only a suppuration in the throat but also a disease with systemic resonance and significant lethal potential.

## Introduction

Cervical suppurations represent an emergency pathology, with a dramatic evolution in the absence of adequate treatment. There is a medico-legal implication of these cases. It is worth noting the lack of a diagnostic and treatment standard, as these cases are quite rare, and unification of diagnostic and treatment protocols has not been achieved [1].

The anatomical substrate for the development of these deep cervical suppurations is represented by the cervical fascia and spaces. Depending on the involvement of these cervical spaces and the risk of compromising the airway or vital structures, cervical suppurations have been divided into low-, moderate-, and high-severity suppurations. Low-severity suppurations (low risk to the airways or vital structures) affect the vestibular, superiostal, body of the mandible, intraorbital, and buccal spaces. Suppurations of moderate severity (moderate risk to the airways or vital structures) involve the submandibular, submental, sublingual, pterygomandibular, submasseteric, superficial temporal, and deep temporal spaces. High-severity suppurations (high risk to the airway or vital pathways) involve the lateropharyngeal, retropharyngeal, pretracheal, prevertebral risk space, mediastinum, and intracranial spaces [2].

The clinical, pathological situations most frequently associated with the appearance of a diffuse cervical suppuration are represented by neglected old dental septal foci (untimely treated and without antibiotic protection), tonsillar pathology, ulcero-necrotic pultacea tonsillitis, neglected or untimely drained peritonsillar phlegmon and without antibiotic protection, traumas represented by embedded pharyngoesophageal foreign bodies, assaults and self-aggressions with blunt objects, and narcomania with the injection of illegal drugs directly into the internal jugular vein [3]. In the evolution of these pathological conditions towards a cervical suppuration, especially important is the presence of diabetes as a favorable condition for sepsis, hematological conditions associated with leukopenia, HIV (human immunodeficiency virus) coinfection, and chronic hepatitis. Social status can favor the appearance of cervical suppurations through a poor nutritional status, with an impact on general and local immunity, through greater exposure to microbial agents, poor oral hygiene with the amplification of saprophytic flora, ignoring dental outbreaks and delaying the initiation of antibiotic therapy, and delaying presentation to the emergency room/doctor in case of the onset of a cervical suppurative pathology [4].

The diffuse cervical suppurations can progress to necrotic cervical fasciitis, a polymicrobial infection with the most common oropharyngeal or odontogenic starting point, with rapidly progressive, destructive evolution in the deep fascial planes of the neck [5].

Progression of necrotizing cervical fasciitis to the mediastinum produces necrotizing descending mediastinitis, which is frequently fatal and has a poor prognosis. Necrotizing cervical fasciitis with necrotizing descending mediastinitis has a 49% mortality rate. Unfortunately, the recent literature does not provide much more optimistic results. For the diagnosis and complex multidisciplinary treatment of mediastinal cervical suppurations, it is also important for the head and neck surgeon to know the pathogenesis of these suppurations and the criteria for early diagnosis to quickly request a thoracic surgery consultation and to approach the case in a multidisciplinary way by including a head and neck surgeon, thoracic surgeon, anesthesiologist, imaging specialist, infectious disease specialist, anatomopathologist, and so on [6].

The mechanisms of the spread of infection from the neck to the mediastinum in these cases are represented by the absence of barriers in the deep fascial planes of the neck, negative intrathoracic pressure, the mechanical and chemical action of gas bubbles produced by anaerobic flora, and vascular septic thromboses that cause tissue necrosis. The extension of cervical suppurations to the mediastinum is done by three major pathways of cervicomediastinal propagation: (1) the pretracheal pathway to the anterior mediastinum, (2) the parapharyngeal or jugulocarotid path, to the middle mediastinum, and (3) the retropharyngeal pathway containing the risk space, to the posterior mediastinum [7].

The treatment of cervicomediastinal suppurations, especially the surgery of these suppurations, requires an understanding of the pathways of propagation and knowledge of the anatomy of the fascial planes and cervical spaces [8].

Necrotizing fasciitis (NCF) is more common in the extremities of the perineum and trunk. Less than 5% of these are cervicofacial. Descending necrotizing mediastinitis (DNM) complicates more than 40% cases of NCF. Mortality of NCF with DNM versus NCF alone is, on average, double. DNM triples the risk of developing septic shock [9].

The diagnostic criteria for DNM presuppose clinical evidence of an oropharyngeal or odontogenic infectious focus, the characteristic imaging appearance of mediastinitis, documentation of necrotizing mediastinal suppuration intraoperatively or at autopsy, and establishing the relationship between DNM and the oropharyngeal or odontogenic focus [10].

Imaging criteria suggestive of mediastinitis on front and profile chest X-rays are represented by widening of the mediastinum and mediastinal emphysema, hydro-aerial levels projected on the mediastinal shadow, unilateral or bilateral pleural effusion, widening of the cardiac silhouette, anterior displacement of the tracheal air column by prevertebral opacity of soft tissues, and the disappearance of lordosis of the cervical spine. The following imaging criteria suggestive of mediastinitis on cervicothoracic MRI examination are essential for early diagnosis: soft tissue infiltration, with increased density of mediastinal fat, mediastinal emphysema and mediastinal fluid collections, with or without air bubbles, and pleural or pericardial effusions [11].

Depending on the data from the chest computed tomography (CT) scan, three types of DNM mediastinitis are described: type I, with infection located in the upper mediastinum and above the tracheal bifurcation; type IIA, the infection extends to the lower and anterior mediastinum; and type IIB, the infection extends to the lower, anterior, and posterior mediastinum [12].

The evolution and prognosis of cervicomediastinal suppurations are burdened by prolonged hospitalization in the intensive care unit (ICU) and in the hospital, with the need for surgical reinterventions in 33% to 100% of cases. The mortality of NCF with or without DNM remains very high, 15-50%, despite intensive treatments. Mortality is associated with late diagnosis and insufficient drainage [13].

Cervicothoracotomy in successive operating times on the same day is associated with lower mortality than cervicotomy and thoracotomy performed separately on different days. Preoperative failure of two or more organs is associated with higher mortality. Common causes of death are sepsis with multiple system organ failure (MSOF), bleeding, aspiration, emphysema, and pericardial empyema with cardiac tamponade [14].

Conditions for improved prognosis are represented by early diagnosis with emphasis on clinical suspicion, early aggressive drainage, massive broad-spectrum antibiotic therapy, and intensive case management [15].

## Materials and methods

This is a retrospective clinical study carried out on 26 cases diagnosed and treated between September 2013 and September 2018 in the ENT (Ear, Nose, and Throat) Clinic Department of the Bucharest University Emergency Hospital (BUEH).

The inclusion criteria in the retrospective analysis were as follows: patients with deep cervical suppurations, patients operated in the ENT Clinical Department of the BUEH, including patients with previous surgical interventions, patients admitted from the ED (Emergency Department) or by intra- and inter-hospital transfer, patients aged more than 18, and patients signing the informed consent.

Exclusion criteria were as follows: patients with localized cervical suppurations (cervical or facial abscesses, superficial cervical collections), patients aged less than 18, and patients who did not sign the informed consent. The therapeutic protocol that we applied to all these patients consisted of a complete set of blood tests (blood count, erythrocyte sedimentation rate, fibrinogen, C-reactive protein including presepsin and procalcitonin, complete biochemistry); cervical and thoracic CT, with contrast agent, to rule out arterial or venous thrombosis; cervicotomies and thoracotomies; admission of patients to the anesthesia and intensive therapy unit after surgery; control cervical and thoracic CT at 48-72 hours postoperatively; and surgical revisions.

The statistical analysis was performed using Excel and PSPP version 2. We did not have a sample size calculation as this is a retrospective descriptive cohort study that included all the cases in the time frame that met the inclusion criteria. The parameters studied included distribution by sex, age groups, social status, clinical picture at presentation, focus/cause of suppuration, comorbidities/risk factors, types of diffuse cervical suppuration, time interval after the onset of symptoms and admission/cervicotomy, bacteriological examination of the wound, number of cervicotomies in our unit, length of hospitalization, and evolution. We performed a separate analysis of NCF case distributions.

## Results

**Figure 1:**
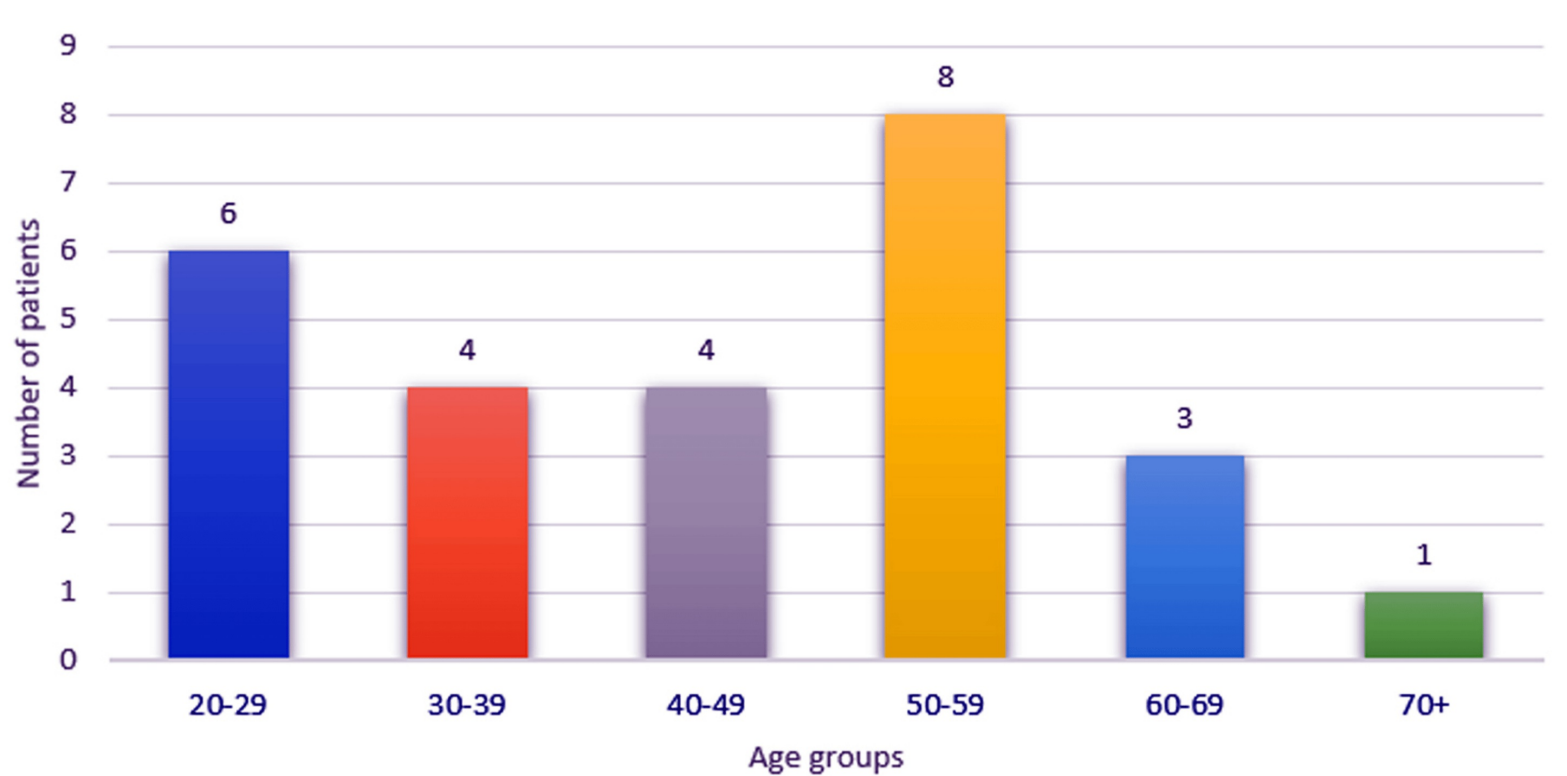
Chart depicting the distribution of the study group by age.

Patients were distributed with a slight male predominance, with 15 males and 11 females (57.69% male). Ages were distributed between 21 and 72 years, with a mean of 46.5 years. The most numerous age group represented was the 50- to 59-year group (eight cases), followed by the 20- to 29-year group (six cases) (Figure 1).

**Figure 2:**
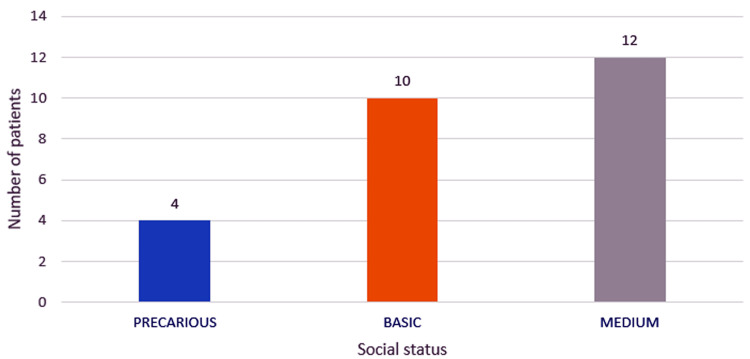
Chart depicting the distribution of the study group by social status.

More than half of the patients (53.84%) had a precarious and modest social status, considering the average wage/economy as monthly income for normal social status, the minimum wage for the economy for modest social status, and the lack of a constant monthly income for precarious status (Figure 2).

**Figure 3:**
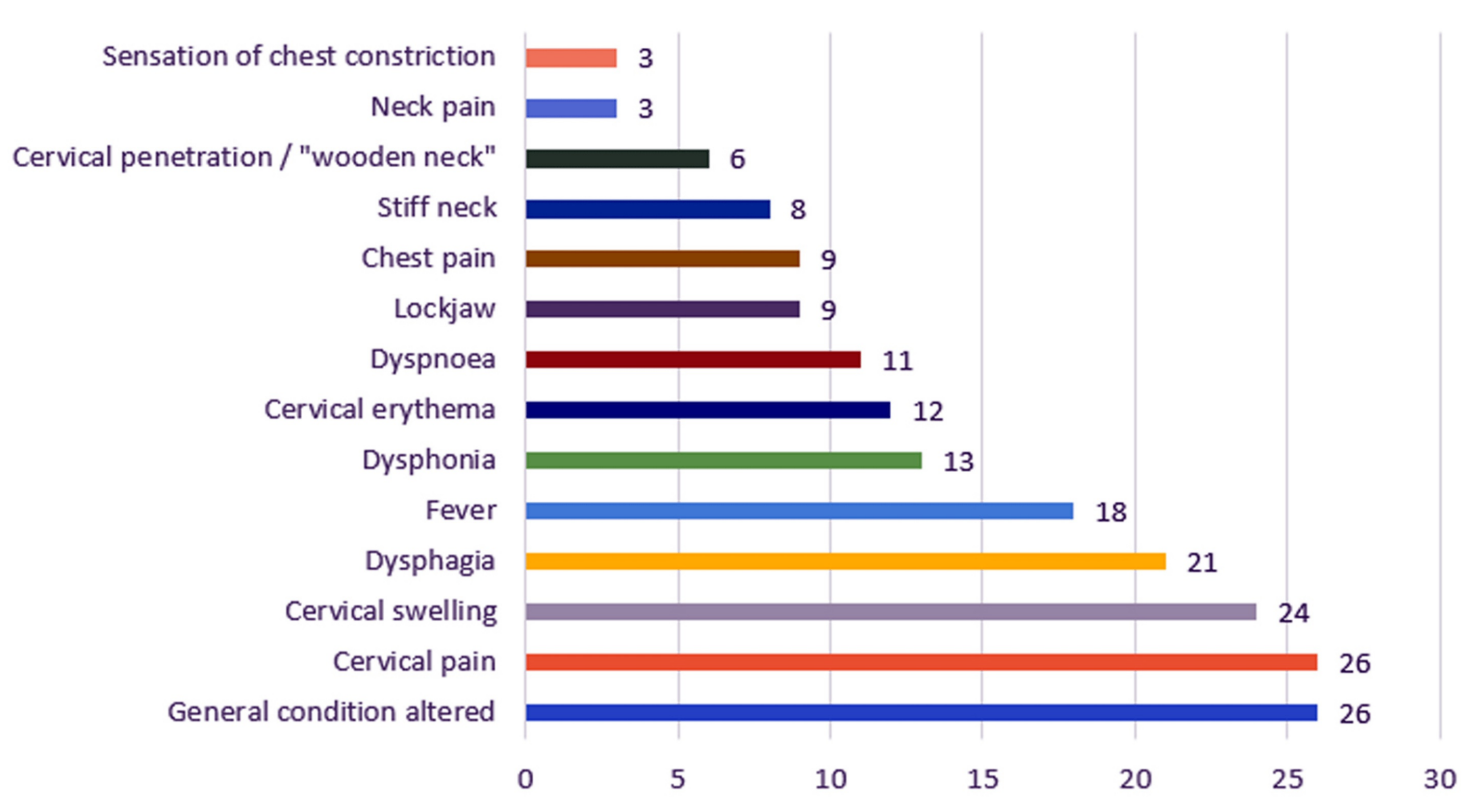
Chart depicting the distribution of the study group according to clinical symptoms.

The clinical picture at presentation of the patients included the following: altered general condition (26 patients, 100%), cervical swelling (24 patients, 92.3%), cervical pain (26 patients, 100%), dysphagia (21 patients, 80.76%), dysphonia (13 patients, 50%), dyspnea (11 patients, 42.3%), fever (18 patients, 69.23%), neck stiffness (three patients, 11.53%), trismus (nine patients, 34.61%), torticollis (eight patients, 30.76%), cervical erythema (12 patients, 46.15%), chest pain (nine patients, 34.61%) sensation of chest constriction (three patients, 11.53%), and cervical infiltration - neck of wood (six patients, 23.07%) (Figure 3).

**Figure 4:**
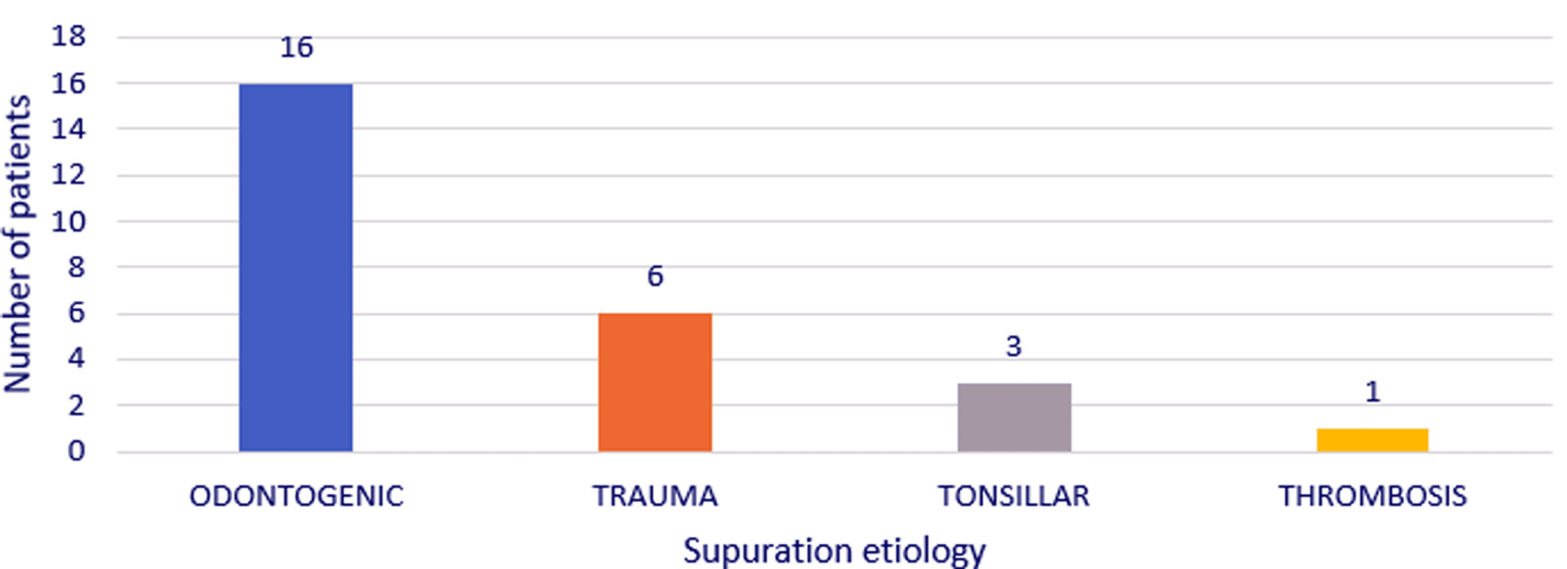
Chart depicting the distribution of the study group by etiology of the suppuration.

The source of cervical or cervicomediastinal suppuration was odontogenic (16 cases, 61.53%), traumatic (six cases, 23.07%), tonsillar (three cases, 11.53%), and septic internal jugular vein thrombosis (one case, 3.84%) (Figure 4).

**Figure 5:**
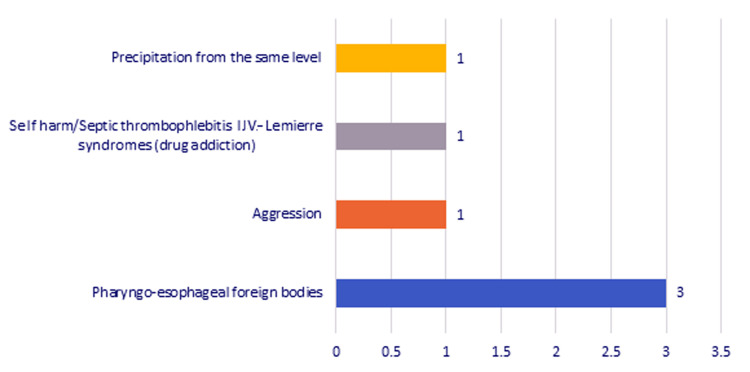
Chart depicting the distribution of the cases with traumatic etiology. IJV, internal jugular vein

Practically, 61.53% among the deep cervical suppurations were odontogenic and 23.07% were traumatic. In this subcategory, of cases with traumatic etiology, patients had pharyngoesophageal foreign bodies (three cases, 11.53%), physical aggression (one case, 3.84%), self-aggression (one case, 3.84%), and precipitation from the same level, and the case of septic thrombophlebitis of the internal jugular vein (Lemierre syndrome) was represented by a case of drug addiction (Figure 5).

**Figure 6:**
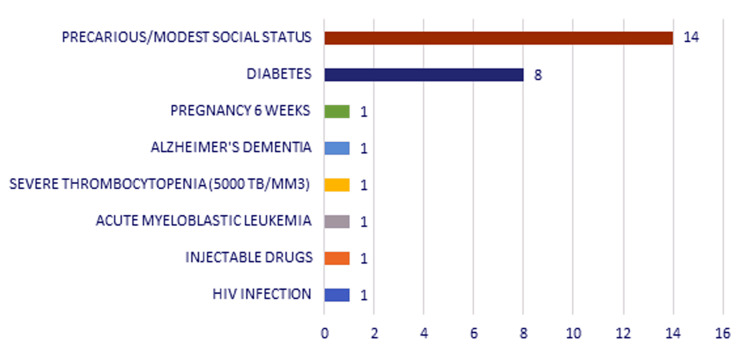
Chart depicting the distribution of the study group according to comorbidities/risk factors.

Comorbidities or risk factors in these patients were represented by diabetes mellitus (eight cases, 33.8%), HIV infection (one case, 3.84%), injectable drugs (one case, 3.84%), acute leukemia (one case, 3.84%), severe thrombocytopenia (one case, 3.84%), Alzheimer's dementia (one case, 3.84%), pregnancy (one case, 3.84%), and precarious and modest social status (14 cases, 53.84%). We consider it worth mentioning that 11 (42.3%) patients had no personal pathological history (Figure 6).

**Figure 7:**
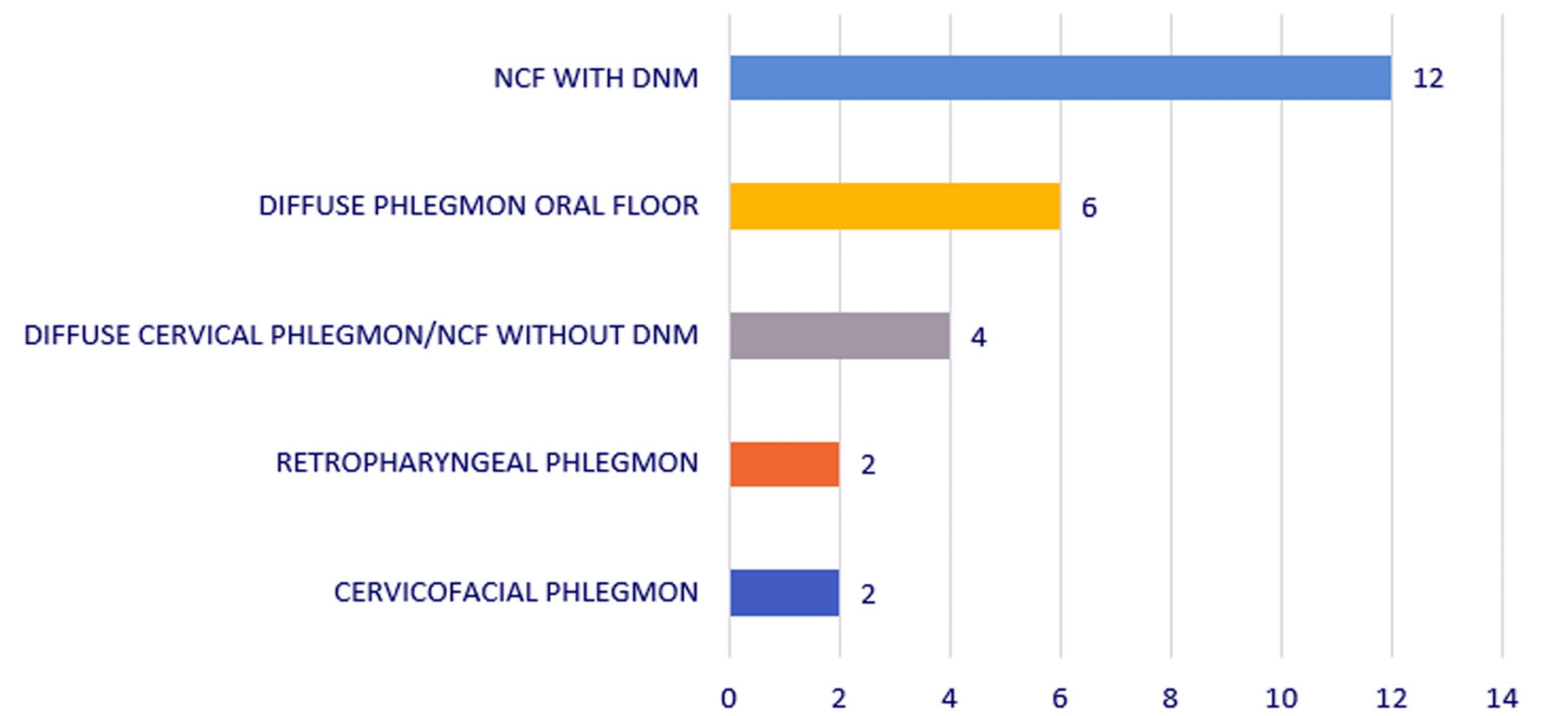
Chart depicting the distribution of the study group by type of suppuration. NCF, necrotizing fasciitis; DNM, descending necrotizing mediastinitis

The types of diffuse cervical suppuration were represented by diffuse phlegmon of the floor of the mouth (six cases, 23.07%), cervicofacial phlegmon (two cases, 7.69%), retroesophageal phlegmon (two cases, 7.69%), necrotizing cervical fasciitis (diffuse cervical phlegmon, four cases, 15.38%), and NCF with DNM (12 cases, 46.15%). In summary, 46.15% of cases with diffuse cervical suppurations were actually cervicomediastinitis, and 61.53% of these suppurations were NCF (Figure 7).

**Figure 8:**
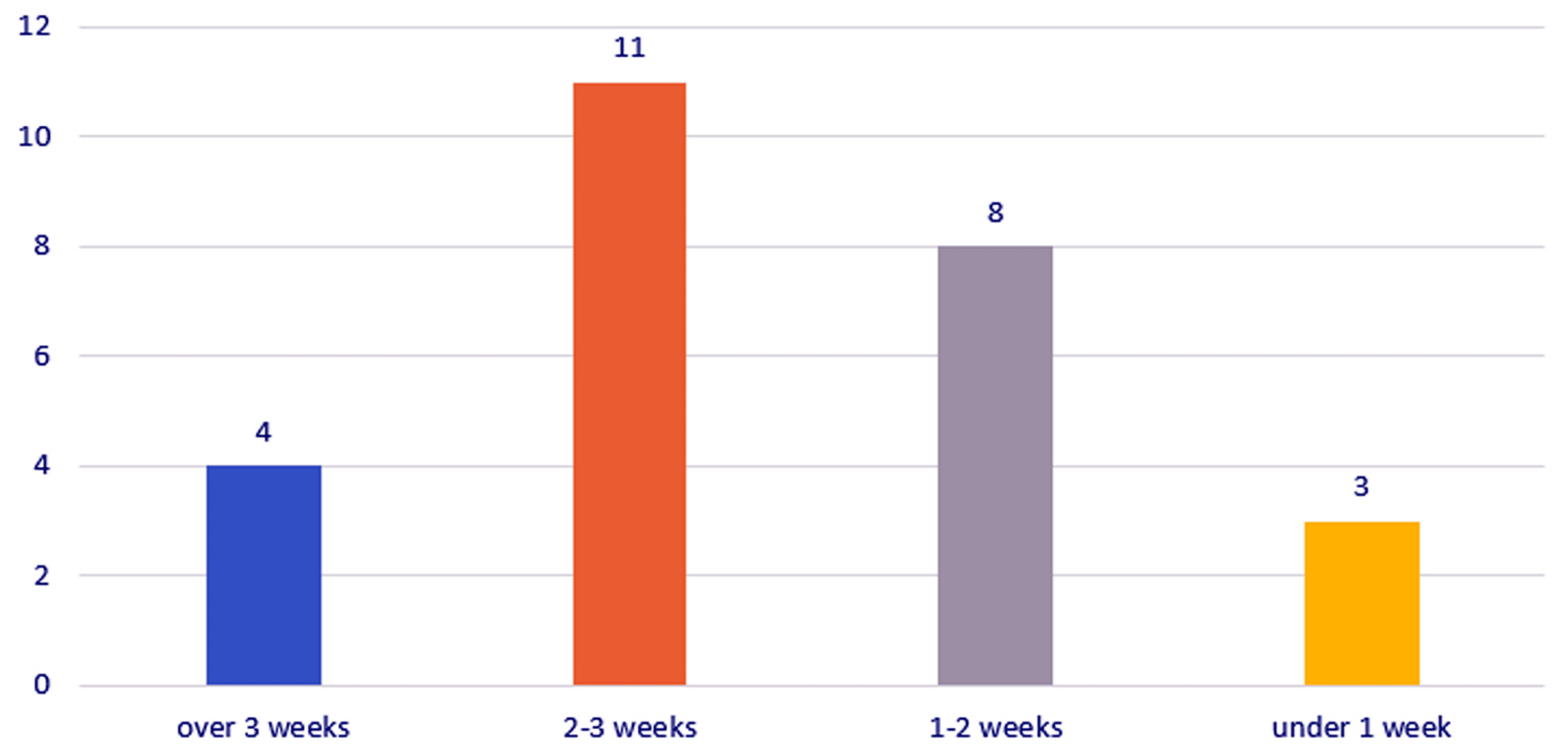
Chart depicting the distribution of the study group by time interval between clinical onset and admission/cervicotomy.

The time interval between clinical onset and admission/cervicotomy was less than one week (three cases), one to two weeks (eight cases), two to three weeks (11 cases), and more than three weeks (four cases). Previous surgical drainage interventions were registered in nine (34.61%) cases (Figure 8).

**Figure 9:**
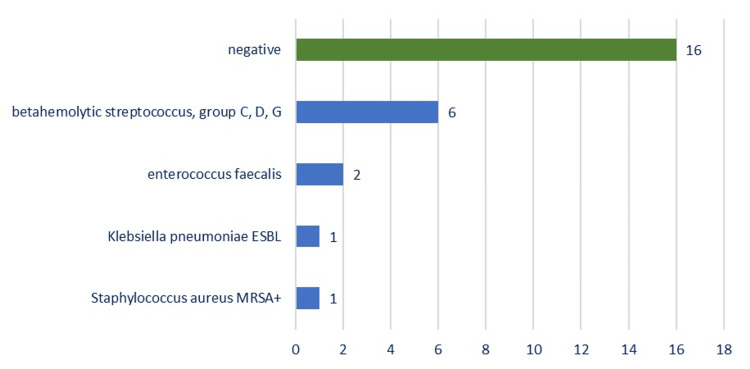
Chart depicting the distribution of the bacteriology results from the supuration swabs. ESBL, extended-spectrum β-lactamase; MRSA, methicillin-resistant Staphylococcus aureus

Bacteriological examination of the wound identified betahemolytic streptococcus groups C, D, and G (six cases), Enterococcus faecalis (two cases), *Staphylococcus aureus* MRSA+ (one case), and *Klebsiella pneumoniae *extended-spectrum β-lactamase (ESBL) (one case). The cultures of the intraoperative samples were negative in 16 (61.53%) cases, almost two-thirds of the cases. It should be noted that in 19.3% of cases, the cultures were positive (Figure 9).

**Figure 10:**
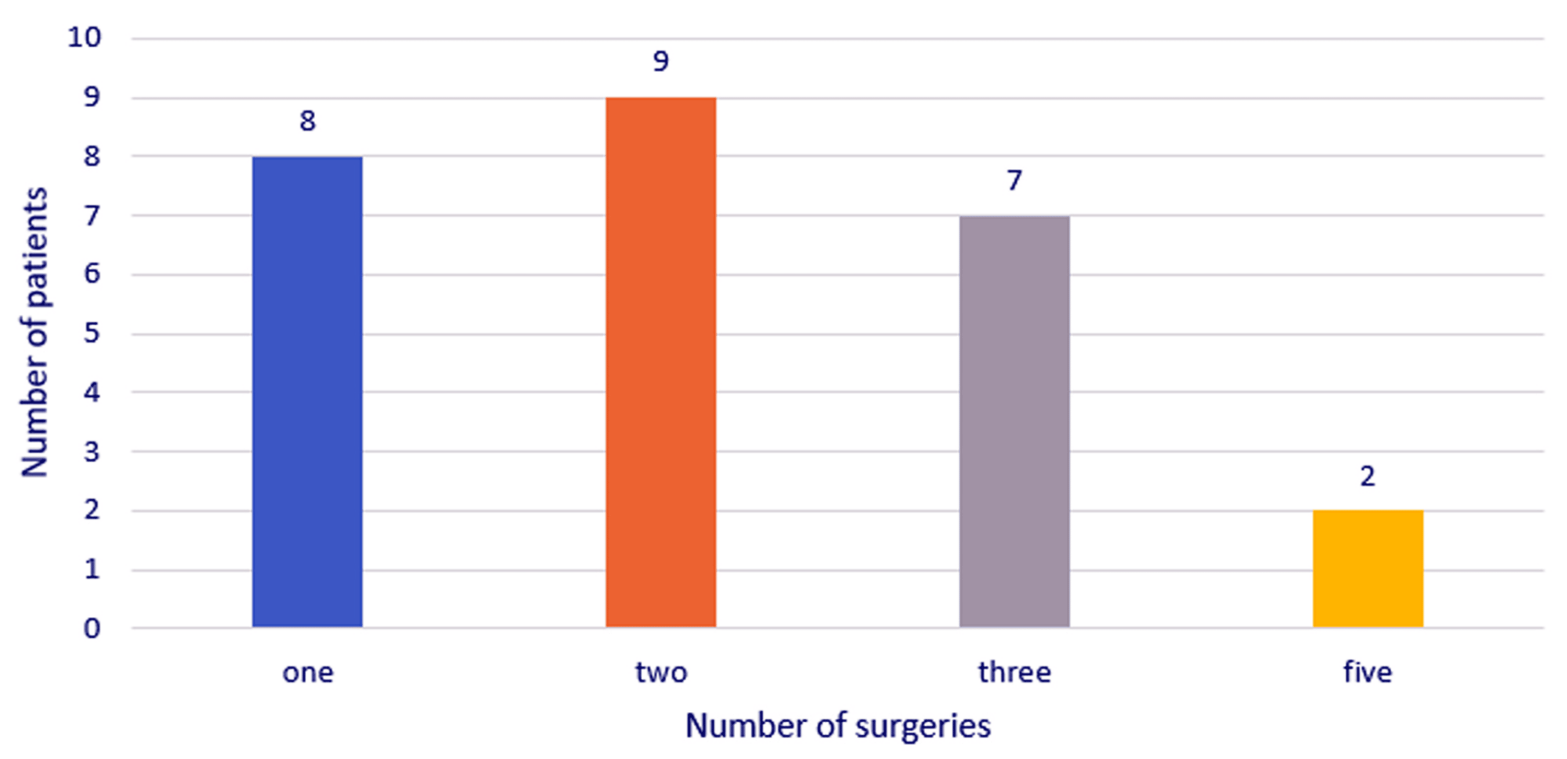
Distribution of the study group by the number of surgeries performed during hospital stay.

The number of cervicotomies performed on patients in the ENT Clinical Department of Bucharest University Emergency Hospital was as follows: one in eight cases, two cervicotomies in nine cases, three cervicotomies in seven cases, and five cervicotomies in two cases (Figure 10).

**Figure 11:**
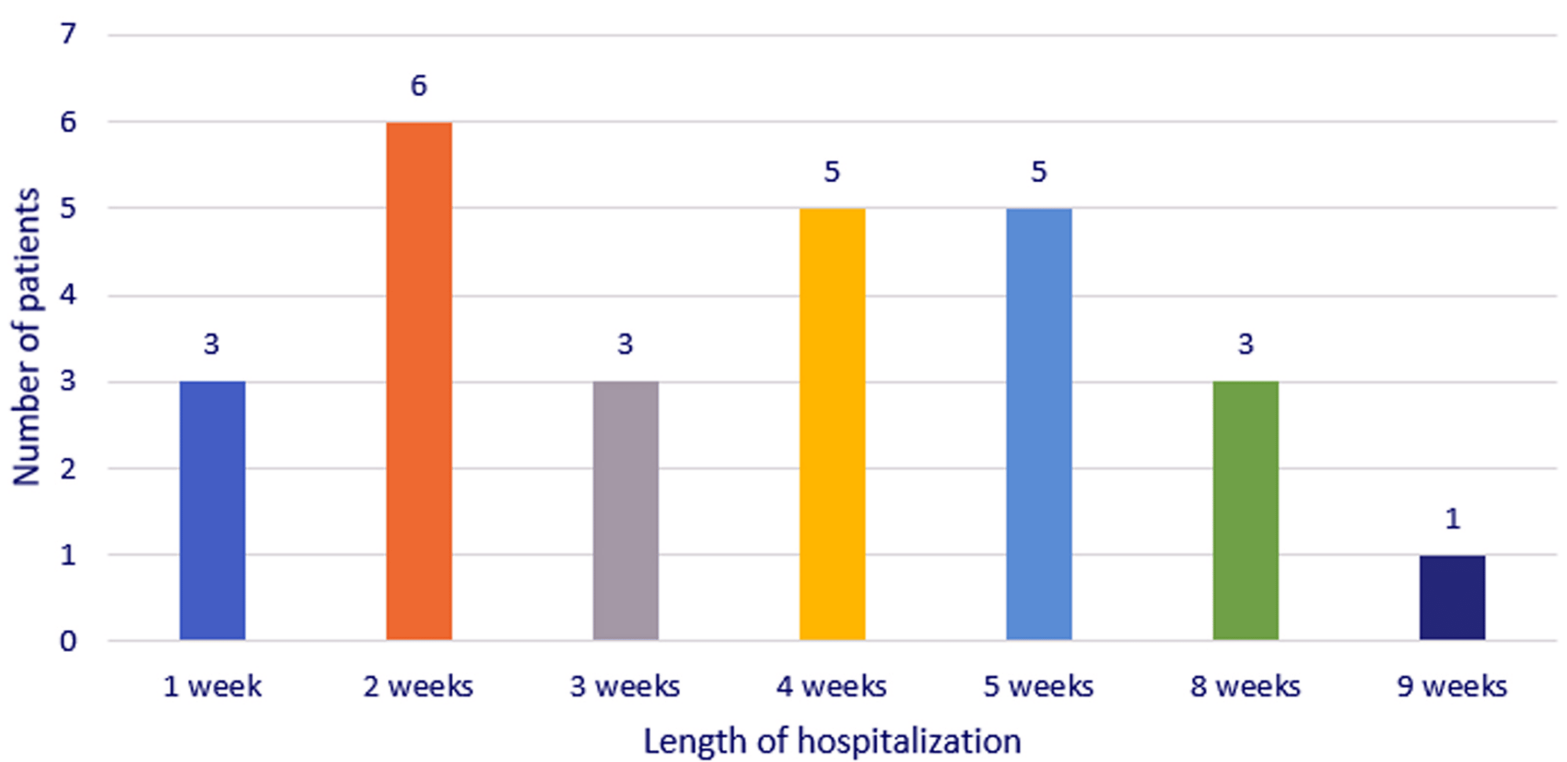
Chart depicting the distribution of the study group according to the length of hospitalization.

The average duration of hospitalization of the patients was 28.26 days (between 3 and 85 days). We had a case with a hospital stay of more than 12 weeks, a 23-year-old young patient with acute suppurative cervicomediastinitis, with involvement in the suppurative process of the supraclavicular fossa, with multiple cervicotomies and drainage thoracotomies, who, in the end, had favorable evolution, with healing (Figure 11).

Among the 26 patients, 20 were declared cured and six died, registering a mortality of 23.07% in our group; therefore, practically one-fourth of cases were of death.

**Figure 12:**
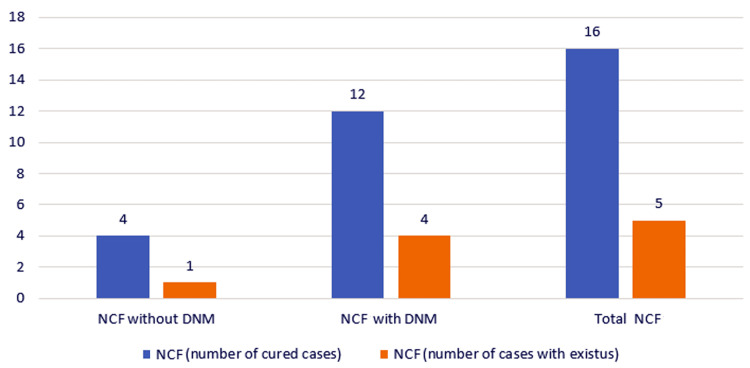
Chart depicting the distribution of the study group according to the final prognosis. NCF, necrotizing fasciitis; DNM, descending necrotizing mediastinitis

Regarding NCF cases, there were four cases of NCF without DNM and 12 cases of NCF with DNM. From the first category, one patient died (25%), and from the second category, four patients died (33.33% mortality). Summing up, out of the 16 cases with NCF, five died, corresponding to a mortality of 31.25% (Figure 12).

## Discussion

Synthesizing our clinical experience, spanning more than two decades, and the diagnostic and treatment algorithms of deep cervical suppuration published in the literature, we propose a 10-step protocol for dealing with a cervical suppuration admitted to the Emergency Room (ER): ENT, oromaxillofacial (OMF), and thoracic surgery consultations, with the aim of identifying the outbreak; determining the severity of suppuration (risk suppuration), at this stage the airway risk and anatomic location are analyzed and imaging assessment is performed by cervical and thoracic CT; evaluating the comorbidities; determining the department for admission (thoracic surgery, ENT, ICU); preoperative administering of broad-spectrum antibiotic treatment/support vital functions; emergency surgical treatment in the first 24 hours after admission, cervicotomy ± thoracotomy; eradicating the outbreak at the same time as the cervicotomy; intensive therapy; careful monitoring (clinical, biological, CT) every 48-72 hours; and surgical revisions.

We can summarize our experience in a tertiary center, describing five important aspects for success in the fight against cervical/cervicomediastinal suppurations, as follows: correct early diagnosis, "moderately aggressive" early surgical treatment, antibiotic treatment, intensive therapy, and close monitoring and nursing.

**Figure 13:**
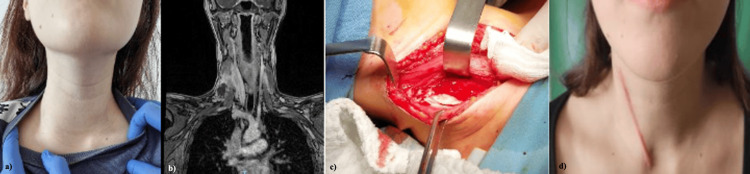
Deep cervical suppuration in a 17-year-old female patient after a viral episode of acute pharyngitis, with an altered immune terrain of autoimmune type. (a) Clinical picture. (b) MRI presurgical imaging. (c) Intraoperative appearance with right cervicotomy. (d) Final aspect at two weeks after discharge (our case).

Early diagnosis requires a correct and complete assessment of the injuries, as well as the identification of comorbidities and risk factors (it should be noted that diabetes was associated in 30.8% of cases and represents a negative prognostic factor in the evolution of suppuration cases cervical) [16]. After performing ENT, OMF, and thoracic examination, ICU, infectious diseases, etc., as well as performing cervical and thoracic imaging, it must be determined if we need to perform emergency surgery. Once diagnosed, the patient is admitted to the ICU, where sepsis management begins. In some cases, autoimmune disease can be a trigger also due to the immunosuppression that the specific medication is inducing (Figure 13).

CT scans must be available as soon as possible for a correct preoperative planning to prevent supplementary lesions to other functional anatomic structures.

In the framework of early surgical treatment, the first stage in the management of such a case refers to the management of the airways, with a choice between intubation and tracheotomy. In most cases, the patient is conscious, cooperative, with difficult intubation conditions. Awake orotracheal intubation is often performed using a bronchoscope or a video laryngoscope, and there are situations in which nasotracheal intubation is performed (for example, in case of very pronounced trismus) or endotracheal intubation is performed, when emergency tracheotomy is needed [17]. In most cases, we would perform the tracheotomy of necessity at the end of the drainage cervicotomy. In our experience, we have found it beneficial for the patient to delay tracheotomy for 24-48 hours in severe suppurations to prevent pulmonary suppurations by draining fetid secretions past the tracheal cannula balloon directly into the trachea. We have had cases of cervical suppuration in which tracheotomy was not necessary, but orotracheal intubation was maintained for three to four days. Among our 26 cases, we practiced tracheotomy of necessity in 15 (57.69%) cases.

**Figure 14:**
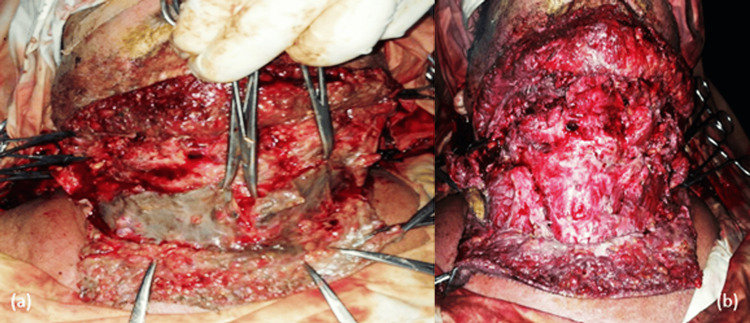
Intraoperative aspects of NCF of odontogenic origin with drainage cervicotomy (a) in the shape of the letter H and extensive debriding (b). NCF, necrotizing fasciitis

The goals of early surgical treatment are drainage and debridement; the cervicotomy must be wide (in L shape, in H shape, in U shape from gonion to gonion); cervicotomy must allow drainage and lesion control, must allow dissection up to the mediastinum, initially/posteriorly, and must be compatible with emergency tracheotomy (Figure 14).

As for debridement, in diffuse cervical suppurations and especially NCF, we are talking about necrotomies. From our point of view, they must be performed moderately aggressively because there is a risk of bleeding that is difficult to control due to septic infiltration of the vascular sheaths. Also, the brutal surgical approach, with intraoperative bleeding, favors septic dissemination; therefore, in our experience, we recommend a careful hemostasis and continuous, gentle washes with betadine serum with the objective of abolishing the septic compartments [18].

**Figure 15:**
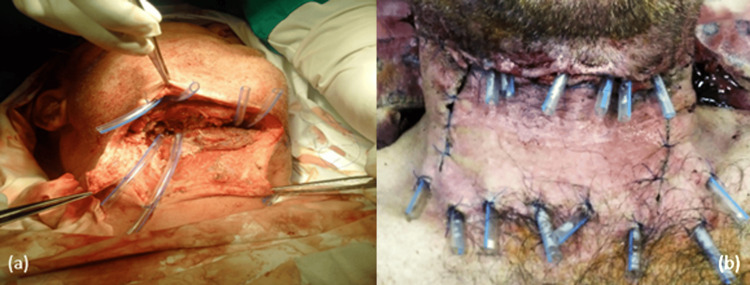
Intraoperative aspects, with drainage systems with rubber tubes in a diffuse phlegmon of the floor of the mouth (a) and in case of NCF (b). NCF, necrotizing fasciitis

In all cases from the studied groups, we kept a a partially closed cervical wound, with sutures to the integument. We performed the drainage of the septic compartments with surgical pledgets soaked in betadine, with rubber blades, as well as with a system of drain tubes for washing/drainage. For the prevertebral space, we prefer prefabricated rubber blades or blades made intraoperatively from sterile rubber gloves to avoid decubitus injuries produced by tubes on large vessels (Figure 15).

Open wound patients were dressed daily in the ICU. We maintained sterile conditions similar to those in the operating room. After removal of the sutures, lavage was performed with betadine, plain or in 50% dilution. There have been cases where we have applied rifampicin powder or iodized boric acid to the wound, but we have no studies that clearly certify the benefit of these applications of antibiotics or antiseptic powder in wounds with cervical suppuration. At 48-72 hours after the follow-up CT, we performed a wound revision with supplementary necrotomies to guide healing. In two cases, we used negative pressure devices, which hastened surgical healing [19].

**Figure 16:**
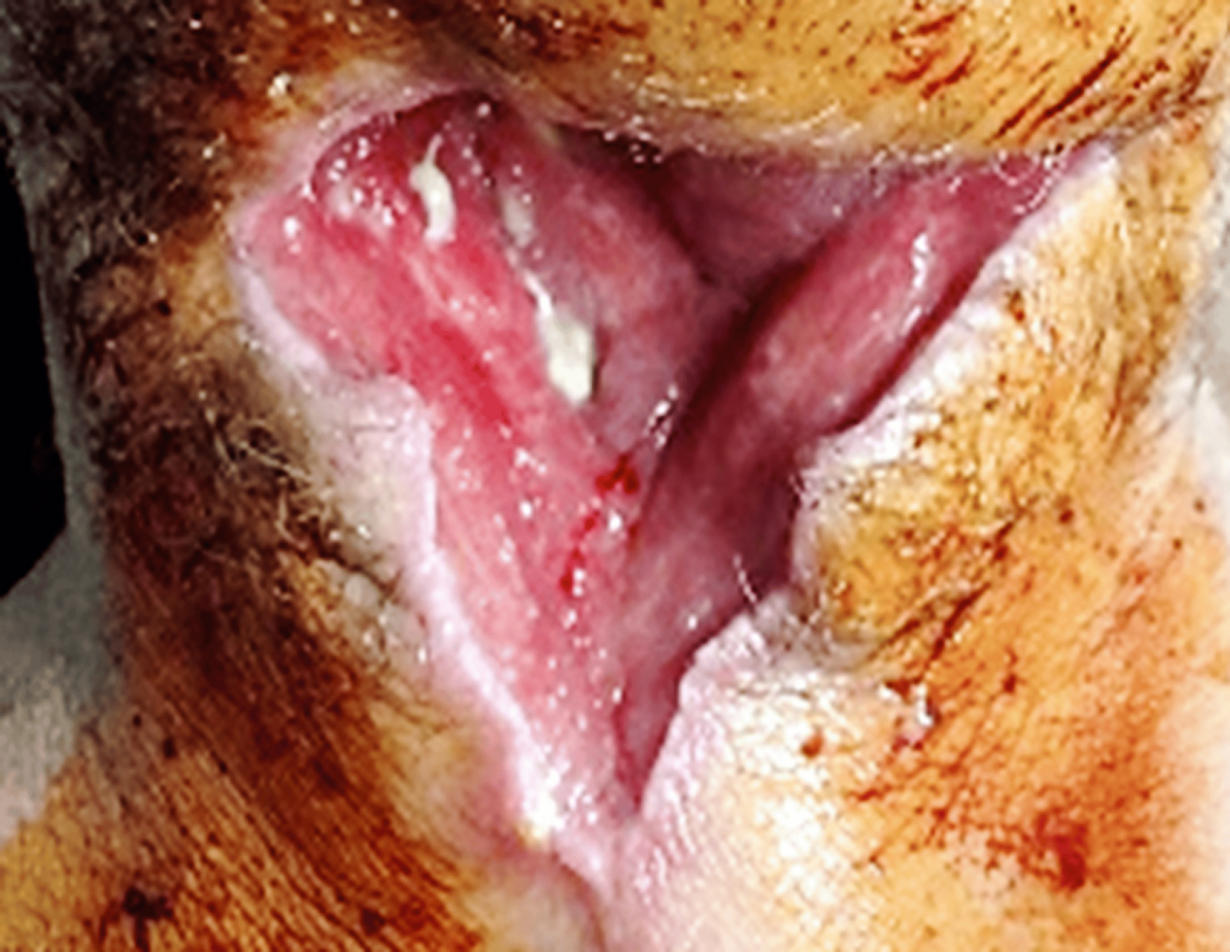
Appearance of vicious scarring, with integumentary substance defect and granulation appearance of the healing wound in a case with diffuse cervical suppuration on the 26th day of hospitalization.

There were also cases where we had to perform plasty of skin defects (Figure 16) and observe the scarring (most often, two to three weeks after the drainage surgery).

The antibiotic should be instituted first to prevent surgical infection. The antibiotic spectrum should cover gram-positive cocci, gram-negative bacilli, and anaerobic bacteria. The antibiotic should be administered pre-, intra-, and postoperatively [20].

The antibiotic combinations we used were based on current guidelines and were also modeled in collaboration with the hospital's infectious disease specialist. Thus, we used penicillin G 24 MU + metronidazole 2 g/day, meropenem 2 g/8 hours + vancomycin 2 g/day + penicillin g 20 MU/day, and piperacillin + tazobactan 4.5 g/6 hours + vancomycin 2 g/ day + penicillin g 20 MU/day. Antibiotics were used for three to four weeks. In penicillin-resistant cases, we used clindamycin. Patients received combinations of antibiotics for 7-10 days, with revision depending on repeated cultures from the wound. The antibiotic treatment was stopped when the patient was transferred from the ICU to the ENT ward. [21].

We also took into account the fact that 61.53% of the intraoperative cultures were negative; therefore, we used these previously mentioned empirical associations, taking into account the saprophytic flora of the oral and pharyngeal cavities. Thus, in the oral cavity, saliva, and dental plaque, we find *Streptococcus*, *Veillonella*, *Corynae bacterium*, *Actinomyces*, *Fusobacterium*, *Rothia*, *Prevotella*, *Neisseria*, and *Haemophilus phorfyromonas*; in the palatine tonsils, we frequently find *Streptococcus neisseria*, *Prevotella*, *Haemophilus*, *Porphyromonas*; and in the esophagus, we find *Streptococcus viridans*, *Fusobacterium*, *Neisseria*, *Haemophilus*, and *Prevotella*. When choosing the combination of antibiotics, we must keep in mind this natural load of saprophytic flora of the oral cavity, pharynx, and esophagus [22].

Intensive care is essential in the management of patients with diffuse cervical suppuration. First of all, it is about the treatment of sepsis and septic shock. We recall that sepsis is the host's systemic response to probable or documented infection. Severe sepsis represents acute organ dysfunction or hypoperfusion secondary to infection. Septic shock consists of severe sepsis + hypotension irreversible on fluids and requiring vasopressors to maintain tissue perfusion. Intensive therapy, therefore, also refers to the support of vital functions. Enteral nutrition is essential and is given via a nasogastric tube or gastrostomy [23].

Nursing these patients is also extremely important in preventing bedsores or other infections associated with the medical act. It is about central and peripheral venous catheters, arterial catheters, sterile dressings, continuous chest lavage, tracheostomy care, and nutrition via nasogastric tube or gastrostomy. Mobilization and psychological counseling in the ICU are also essential, as the patient is most often conscious, with long hospitalization in the ICU, which underlines the severity of the disease and the risk of death [24].

Careful monitoring consists of following some parameters of evolution, such as fever, need for inotropic support, general state/state of consciousness, biological inflammatory syndrome, control imaging (cervicothoracic CT two to three days postoperatively or as needed, interpreted in the context of evolution clinics), and the periodic examination of the wound, together with the thoracic surgeon. Sometimes serial sonographies of the airway can predict the optimal moment for detubating the patient [25].

The evolution of cervicomediastinal suppurations requires a long-term hospitalization, on average three to four weeks, with frequent occurrence of immediate and remote complications. Immediate complications are MSOF, acute respiratory distress syndrome, heart rhythm disorders, cervical/thoracic wound bleeding, salivary fistulas, pleural empyema, pneumothorax, pericarditis, pneumoperitoneum, and peritonitis. Distant complications are represented by MSOF, bronchopulmonary suppurations, upper digestive hemorrhage, tracheal stenosis, vicious cervical scarring, clostridium difficile diarrheal disease, deep vein thrombosis, and anxiety-depressive disorders [26].

**Figure 17:**
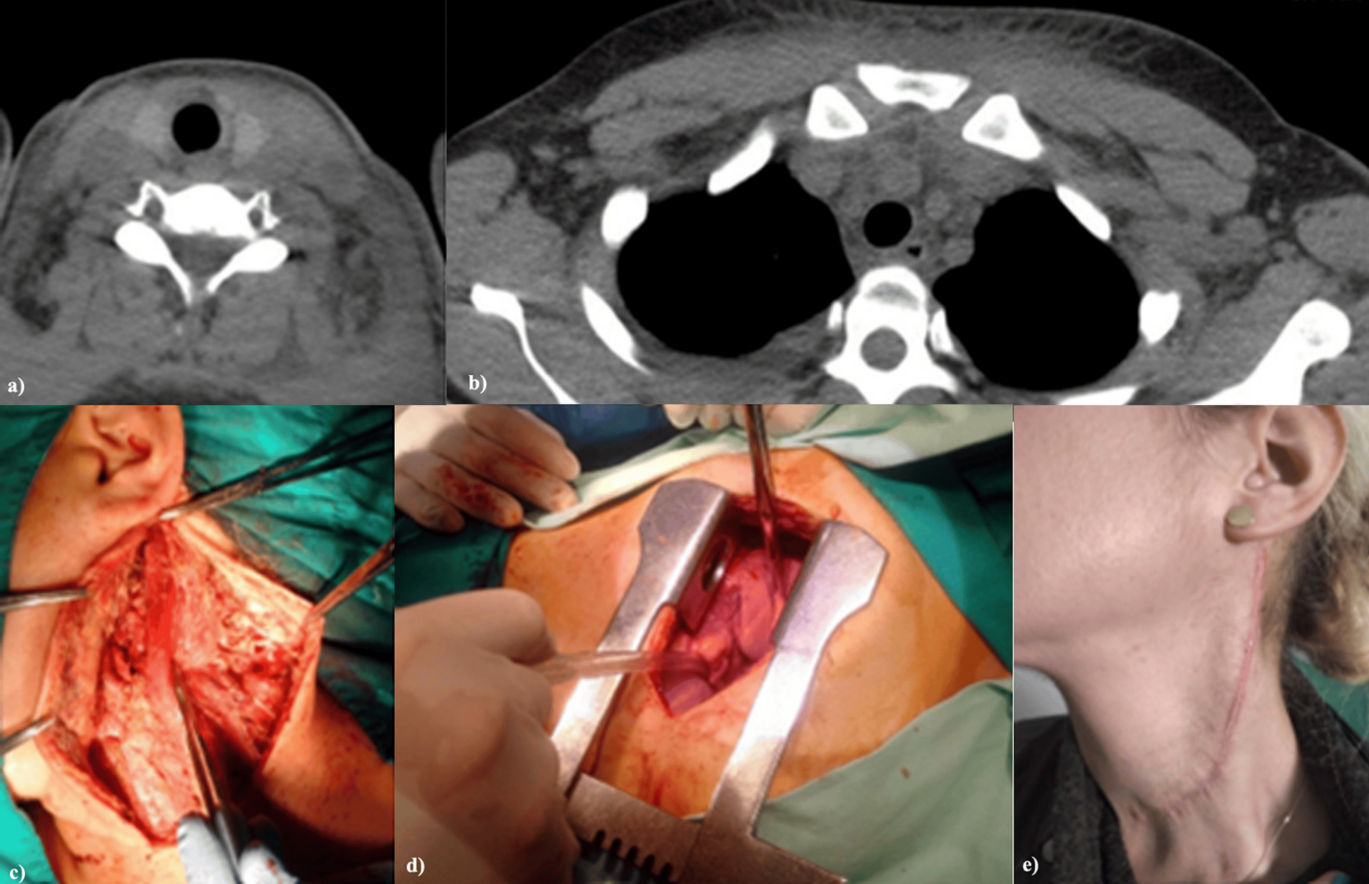
Cervical and thoracic CT appearance and intraoperative findings in a 32-year-old female patient with left cervical hematoma due to aggression, complicated with cervicomediastinitis. (a) Axial CT scan of the neck. (b) Axial CT scan of the thorax. (c) Surgical aspect from the neck. (d) Surgical aspect from the thorax. (e) Final aspect two weeks after discharge.

Positive prognostic factors are early diagnosis, early intervention, outbreak eradication, and nutritional support. Negative prognostic factors are late diagnosis, comorbidities (especially diabetes), age over 50 years, social status, and time interval from symptom onset to surgical intervention (Figure 17).

Cases of cervicomediastinal suppuration can become medico-legal cases in situations of unfavorable evolution, resulting in the death of the patient. From this point of view, it is important to be able to prove that a timely diagnosis was made, an adequate surgical treatment was provided, an adequate antibiotic treatment was provided, intensive therapy was carried out, and, above all, informed consent of the patient was obtained on the procedure therapeutic. It can be useful to have elements that document cases with a possible unfavorable outcome, these can be represented by photographs of the patient intraoperatively (in this case, with the need for signed consent), bacteriological examinations of intraoperative samples, and histopathological examination to document necrosis in the surgical pieces as well as the few histological elements that characterize NCF [27].

A future development of AI-powered diagnostic and management tools could help the clinician in an early and rapid diagnosis and management of such complex and interdisciplinary case of cervical suppurations [28].

The limitations of the present study are derived from the limited number of cases. These are cases from a single-center experience, although they are very complex cases requiring an interdisciplinary approach. Such cases are referred to our hospital because few healthcare facilities have the necessary specialists and equipment to successfully manage these emergency patients. Another limitation is that the informatic system in our unit was changed in 2013 and we could not access older data on the subject. We did not extend our analysis in the COVID-19 pandemic to not introduce other possible sources of bias.

## Conclusions

Deep cervical suppurations and cervicomediastinal suppurations represent a chapter of pathology with major lethal potential. The chances of therapeutic success are increased by a correct diagnosis, early surgical intervention with drainage of the focus that generated the suppuration, broad-spectrum antibiotic therapy, intensive care and nursing, and careful monitoring.

Based on our experience in a tertiary referral center for head and neck surgery, as well as literature reviews on this topic, we propose a 10-step management protocol for the management of a cervical suppuration: ENT consultations, OMF and thoracic surgery, with the aim of identifying the outbreak; determining the severity of the suppuration (suppurations at risk), at this stage analyzing the risk for the airway and the anatomical location and performing the imaging assessment through cervical and thoracic CT; evaluating the terrain and comorbidities; establishing the place of admission (thoracic surgery, ENT, ICU); preoperative institution of broad-spectrum antibiotic treatment/support vital functions; emergency surgical treatment in the first 24 hours after admission, cervicotomy ± thoracotomy; eradicating the outbreak at the same time as the cervicotomy; intensive care; careful monitoring (clinical, biological, CT) at 48-72 hours; and surgical revisions. In a synthetic way, the multidisciplinary approach to these suppurations, including the head and neck surgeon, the thoracic surgeon, the OMF surgeon, anesthetist, imaging specialist in infectious diseases, anatomopathologist, psychologist, and so on, is the key to success in a patient who presents not only a suppuration in the throat but also a disease with systemic resonance and significant lethal potential.
